# Orthodontic retainers: are they all the same?

**DOI:** 10.1590/2177-6709.29.6.e24spe6

**Published:** 2025-01-13

**Authors:** Telma Martins de ARAUJO, Paula Paes FERREIRA, Izabelle Alice Pinheiro Barros LISBOA, Carlos Jorge VOGEL, Carolina Ribeiro STARLING

**Affiliations:** 1 Federal University of Rio de Janeiro, School of Dentistry, Department of Orthodontics (Rio de Janeiro/RJ, Brazil).; 2 Federal University of Bahia, School of Dentistry, Department of Orthodontics (Salvador/BA, Brazil).; 3 Federal University of Bahia, School of Dentistry, Department of Dentistry and Health (Salvador/BA, Brazil).; 4 FAIPE - Higher Education Institution, School of Dentistry, Department of Orofacial Harmonization (Cuiabá/MT, Brazil).; 5 University of São Paulo, School of Dentistry, Department of Orthodontics (São Paulo, Brazil).; 6 University of Illinois, Department of Orthodontics (Illinois /USA).

**Keywords:** Orthodontic retention, Stability, Aging, Dental alignment, Contenção ortodôntica, Estabilidade, Envelhecimento, Alinhamento dentário

## Abstract

**Introduction:**

It is known that the stability of the results obtained with orthodontic treatment depends, in addition to the functional and aesthetic aspects, also on the adequate planning of the retention devices, the patient’s compliance with this new phase, and the physiological changes that the human body experiences over the years, throughout the craniofacial aging process.

**Objective:**

This article discusses the importance of the orthodontic retention phase and the influence of diagnosis, planning and execution of corrective treatment of malocclusions, in order to achieve the expected success.

**Methods:**

Throughout the text, different types of retainers and approaches during this phase will be presented, with the aim of ensuring the stability of the results obtained after correcting problems in the vertical, transverse and anteroposterior directions.

**Results:**

Orthodontic retainers are not all the same. The retention protocol must be performed in an individualized and planned manner, taking the initial dental positions as a reference.

**Conclusions:**

The orthodontist must inform the patient and parents about important aspects of how to maintain the occlusion achieved with orthodontic treatment. The retainers installed after the end of corrective treatment are not everlasting, they can suffer damage with use and must be replaced. Therefore, the patient must be aware of the importance of using retainers as prescribed by the orthodontist, and of returning for scheduled review appointments.

## RESUMO

**Introdução:**

Sabe-se que a estabilidade dos resultados obtidos com o tratamento ortodôntico depende, além dos aspectos funcionais e estéticos, também do planejamento adequado dos aparelhos de contenção, da adesão do paciente a essa nova fase, e das mudanças fisiológicas que o corpo humano experimenta com o passar dos anos, ao longo do processo de envelhecimento craniofacial.

**Objetivo:**

O presente artigo discute a importância da fase de contenção ortodôntica e sobre a influência do diagnóstico, planejamento e execução do tratamento corretivo das más oclusões, para que se alcance o sucesso esperado.

**Métodos:**

Ao longo do texto, serão apresentados diferentes tipos de aparelhos de contenção e condutas durante essa fase, com o objetivo de garantir a estabilidade dos resultados obtidos na correção de problemas nos sentidos vertical, transversal e anteroposterior.

**Resultados:**

As contenções ortodônticas não são todas iguais. O protocolo de contenção deve ser realizado de modo individualizado e planejado, tendo como referência as posições dentárias iniciais.

**Conclusões:**

O ortodontista deve informar ao paciente e responsáveis aspectos importantes sobre como manter a oclusão conquistada com o tratamento ortodôntico. As contenções instaladas após a conclusão do tratamento corretivo não são perenes, elas podem sofrer danos com o uso e devem ser substituídas. Por isso, o paciente deve estar ciente da importância de usar as contenções conforme prescrito pelo ortodontista, e de retornar para as consultas de revisão agendadas.

## INTRODUCTION

Orthodontic treatment consists of two phases, one active and one passive. The active phase involves diagnosis, planning and treatment of the malocclusion - a stage that depends mainly on the technical preparation and competence of the orthodontist, but also on the patient’s cooperation in following the instructions received. The passive phase, applied by the orthodontist, begins with the installation of the retainers, and it is the patient’s responsibility - as regards the use and care of the retainers - to maintain the results achieved with the corrective treatment.

The orthodontist’s main therapeutic goal is to achieve excellent occlusion and aesthetic-orofacial balance, in order to obtain a stable result. However, long-term stability is one of the most challenging factors in orthodontic treatment.^1^ ^-^ ^7^

Post-treatment dental changes can be minimized if: orthodontic diagnosis and planning are correct; the occlusal objectives are achieved, with healthy adjacent tissues; and retainers are used with an appropriate protocol.^8^ ^,^ ^9^ The orthodontist has the challenge of finding the ideal position of the teeth, in their respective bone bases, as pressure from adjacent tissues can cause relapse and unwanted tooth movement.^9^

Although it is not known for sure what causes the lack of stability in orthodontically treated cases, some authors emphasize that treatments completed with excellence are more stable when compared to those that were undercorrected or that underwent dental expansion. Furthermore, success in the professional’s clinical performance lies in the careful reassessment, during the retention period, of the results achieved with the treatment.^3^ ^-^ ^5^ ^,^ ^10^ ^,^ ^11^

Therefore, the objective of this article is to discuss the most relevant aspects related to stability and retention in Orthodontics, based on classical literature, current literature and clinical experience. 

## TERMINOLOGY AND DEFINITIONS

In the literature, there are numerous terms used when the subject addressed is retention. The variety of terminologies for this topic confuses and hinders communication not only between professionals, but also between patients.^12^

Retention is defined as the maintenance of teeth in ideal aesthetic and functional positions.^13^ Relapse would be related to dental changes after orthodontic treatment and, in these cases, the teeth would be returning, partially or completely, to the original malocclusion.^12^ Changes in tooth positions can happen in the short term, due to poorly conducted treatment or non-use of retention devices; or in the long term, occurring even in well-treated cases, as a result of the natural aging of individuals.^6^ ^,^ ^7^ ^,^ ^14^ ^,^ ^15^

Physiological recovery, in turn, is described as the return of normal characteristics and functions. For example, in young patients who underwent early treatment and completed it before reaching skeletal maturity, it is common to observe residual growth, in which genetic characteristics redirect this growth, which has yet to occur, towards its initial characteristics. Developmental changes are related to the aging of the individual. So, regardless of whether treatment has been carried out or not, they will always occur.^12^

## HISTORY

For many years, the retention phase was not considered important to orthodontists. However, the need to understand what happened to make the teeth move over the years, after treatment, aroused the curiosity of some researchers, who began to investigate which factors were related to these changes. Different theories emerged over the years, but this does not mean that each new theory discarded the previous one; on the contrary, they added to each other, adding value ​​to the existing concepts on the subject.^13^

Four schools contributed to the concepts discussed today about long-term stability: the occlusion school; the apical base school; the lower incisors school; and the musculature school. The occlusion school advocated that tooth occlusion is a key factor in long-term stability. The apical base school believed that the relationship of the basal bones is one of the most important factors in correcting malocclusion and maintaining the results achieved, and this gave rise to ideas such as the need to respect the intercanine and intermolar distances, and that the length of the dental arch could only be increased to a certain limit. The lower incisors school said that these teeth should be vertical and well placed in the basal bone. Finally, the musculature school introduced the idea of ​​the need for muscular balance for treatment stability.^13^

The retention was still based on nine basic theorems, namely: 1) teeth that were moved tend to return to their original position; 2) eliminating the causes of malocclusions reduces relapses; 3) overcorrecting malocclusions is a safety factor; 4) excellent functional occlusion is a potential factor for stability; 5) bones and adjacent tissues must have time to adapt to the new positions of the teeth; 6) if the lower incisors are vertical and on the basal bone, they will tend to remain in good alignment; 7) corrections performed during the patient’s growth are less prone to relapse; 8) the more teeth are moved, the lower the chance of relapse; 9) the shape of the dental arch, especially the lower one, must be maintained.^13^

## DENTAL AND SKELETAL PHYSIOLOGICAL CHANGES

Thilander^16^ studied 436 plaster models of 55 individuals, aged 5 to 31, with normal occlusion and not undergoing orthodontic treatment. The following parameters were analyzed: dental arch perimeter, width and length; and palate height. After careful evaluation, it was found that the biggest change in dentition occurs between mixed dentition and permanent dentition (5 to 13 years old). Furthermore, small changes also occur between adolescence and adulthood (16 to 31 years old) - important data that must be considered in the diagnosis, treatment plan and post-orthodontic treatment stability.^16^

With the eruption of permanent incisors, there is an increase in the perimeter of the anterior segment of the dental arch, especially the upper one. When the permanent canines erupt, a slight increase occurs in the intercanine distance, with a consequent increase in the length of both dental arches. On the other hand, in the posterior segment, there is a decrease in the perimeter of the arch, especially in the lower arch, due to the mesial migration of the first molars to the Leeway space of Nance, reducing the intermolar distance and the length of the dental arch.^16^

A slow and gradual decrease in the posterior perimeter of the dental arches occurs between adolescence and adulthood, indicating physiological migration of the occlusion in the anterior direction. In the period evaluated by Thilander,^16^ a slight increase of 1 mm in the total perimeter of the upper arch and a decrease of 4 mm in the lower arch was found. This dentoalveolar development should be considered when planning treatment of patients with anteroinferior crowding, as well as when evaluating stability after treatment.^16^ Changes during the retention and post-retention periods cannot be separated from the individual’s natural aging process, which occurs regardless of whether they have been orthodontically treated or not.^15^

Behrents^17^ examined 524 cephalometric radiographs of 153 individuals belonging to Bolton’s study,^18^ and evaluated the changes through superimpositions of cephalometric tracings. In the aforementioned study, it was found that: considerable craniofacial changes occur beyond the age of 17, until advanced age, in an apparently slow way; the size and shape of the craniofacial complex changes over time; growth in women is smaller at all ages and more vertical than in men; there is rotation of the mandibular plane (counterclockwise in men and clockwise in women); and compensatory changes are noted in the dentition and soft profile.^17^

Many orthodontists prefer to treat their patients early, due to advantages such as: using growth in favor of treatment; less need for tooth extractions or orthognathic surgery; lower risk of iatrogenic events; less need for patient cooperation; and, above all, better and more stable results.^2^ ^,^ ^19^

However, skeletal changes may occur when treatment is completed with the individual in an active growth process.^12^ Many patients are treated in adolescence, creating an opportunity for subsequent growth of the maxilla, mandible and midface.^20^ Genetic characteristics will make residual growth appear to relapse the initial problem, especially in the mandible. However, this type of change is not considered a relapse; but rather the result of unwanted residual growth.^12^

On the other hand, the patient’s growth pattern may be limited, that is, not respond to orthodontic treatment, as occurs in cases with greater facial growth due to vertical excess of the maxilla. These patients have an abnormally long face, which prevents the lips from sealing at rest. This situation causes muscle imbalance and a potential chance of relapse.^9^

### FACIAL AGING AND THIRD MOLARS

It is sometimes curious to observe the appearance or worsening of dental crowding in the region of the lower incisors, whether in patients who were treated orthodontically and did not use the retainers properly, or in individuals who have never undergone any intervention. Richardson^21^ carried out a literature review to compile studies that recorded the changes that occurred in the lower arch from 7 to 50 years of age. In this study, the author highlighted that the greatest dental crowding in the lower arch occurs from 13 to 18 years of age, going through a period of greater stability, and increasing again in the third and fourth decades of life. This variation in lower arch change reflects the dynamics of developmental effects and the multifactorial etiology of long-term crowding.^21^

**Figure 1:**
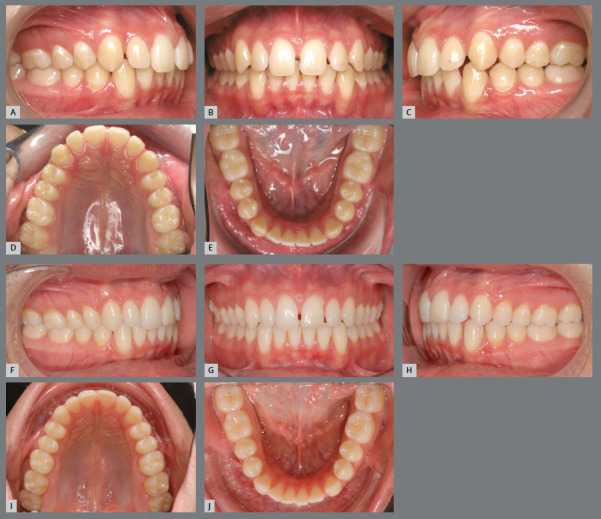
Male patient, 17 years old, with spaces between teeth in the upper and lower arches **(A-E**). When returning 10 years after the first appointment **(F-J)**, without having undergone any orthodontic treatment, it was possible to observe, based on measurements on models: decrease in the spaces between the teeth, and in the length of both dental arches; decrease in transverse dimension of the upper and lower arches (in the canines, first and second premolars); increase in transverse dimension in the second molars; and decrease in overbite.

The factors responsible for lower anterior crowding are diverse, and act alone or together, at different stages of dentition development. Two important points are discussed in the literature: the tendency for mesial inclination of teeth, due to the resultant of masticatory force in this direction; and the presence of a third molar, which can contribute to the mesialization of the lower teeth during its eruption process, if mesially inclined. Furthermore, with age, there is a decrease in the length and width of the upper and lower dental arches (Fig 1), resulting in crowding in the anterior region, if retainers are not used after corrective treatment.^21^

Relapses are multifactorial and there are numerous potential causes; however, most causal factors may be related to the normal developmental process.^22^ Because the dentoalveolar process is part of the craniofacial complex, it is influenced by changes in different parts of the skull;^23^ therefore, it is not possible to guarantee the patient whether these changes will occur or not^15^. Also, when they do occur, they are gradual and physiological and would happen regardless of whether the patient had orthodontic treatment or not.^6^ ^,^ ^24^ ^-^ ^25^

## CASES TREATED WITH OR WITHOUT EXTRACTIONS

The decision about whether or not to extract teeth during orthodontic treatment depends on an accurate diagnosis and is part of the treatment plan of many orthodontists, to keep the teeth within their bone bases, avoiding large expansions and projections. However, “extracting or not extracting”, in isolation, is not enough to maintain the stability of treatment performed.

The literature is controversial regarding the higher rate of tooth crowding in the long term, when comparing cases treated with and without tooth extractions.^12^ ^,^ ^26^ A longitudinal study on a randomized sample conducted by Rossouw^12^ showed that the stability of anteroinferior crowding does not depend on whether the individual was treated with or without extractions, as there was no statistical difference between the groups. Also, the author highlighted that the important aspect for the long-term stability of orthodontically treated cases is the correct diagnosis and planning of each malocclusion, careful orthodontic technique and management during the retention phase.^12^

Factors such as type of malocclusion, sex and age at which the patient started treatment, overbite, overjet, initial and final alignment, width and length of the arch, and duration of retention are not characteristics capable of predicting stability or relapse in the long term of patients treated with premolar extraction. As already mentioned, the length and width of the dental arch decrease over time and, associated with this, the increase in dental crowding after treatment, and the excess of overbite and overjet, as well as the tendency for vertical growth, were the factors most related to relapse.^27^

Little^27^ reported that only 30% of patients treated with premolar extraction had a stable long-term result, as a variation in the maintenance of alignment was observed over the years and was considered unpredictable. Therefore, the author suggests that, for stability to be guaranteed, retention must be used for life.^27^

A study by Woodside et al.^28^ also reinforced the idea that premolars extraction does not influence the maintenance of alignment in the long term. The authors found no statistical difference, regarding the alignment of the anterior teeth, between the group that had serial extraction of four premolars and the group that did not have any extraction. They also reported that the degree of long-term crowding was the same in both groups. However, the authors found that patients with extractions and who also received orthodontic treatment appeared to have more crowding of the lower incisors in the long term.^28^

Even though the indication for incisor extraction is well documented, the study by Little^27^ demonstrated that patients treated with extraction of anterior teeth had greater long-term stability in terms of alignment of anterior teeth than those treated with premolars extraction.^27^ However, the decision to extract a lower incisor should not be based solely on crowding of the anteroinferior segment. Cases in which the extraction of these teeth will eliminate the size discrepancy between the lower and upper anterior teeth (Bolton discrepancy) are the most suitable for lower incisor extraction.^13^

## FACTORS INFLUENCING RELAPSE

### ANTEROPOSTERIOR FACTORS

#### Intercuspation

**Figure 2:**
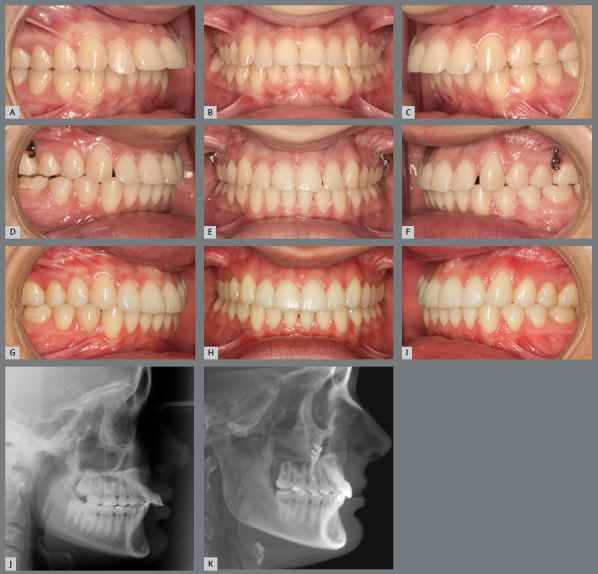
Patient with Angle Class II, division 1, overjet of 10mm **(A-C**). Malocclusion was solved with miniplates for upper arch distalization. Correction of the anteroposterior problem can also be observed, as well as shallow and worn cusps of posterior teeth **(D-**F). In the final photographs, the reconstructed cusps can be observed, in order to provide better intercuspation and greater stability of the anteroposterior relationship, which was achieved by orthodontic treatment **(G-**I). Comparison of the initial and final radiographs**(J,**K) shows the upper arch distalization and correction of the increased overjet.

Posterior intercuspation (i.e., an occlusion with well-fitting cusps in canine, premolar, and molar relationships) contributes to anteroposterior and transverse stability. Also, the adequate relationship of the anterior teeth contributes to anteroposterior and vertical stability.^7^ ^,^ ^29^ ^-^ ^31^ Low or worn cusps should be reshaped with restorative material (Fig 2), aiming to favor the occlusal stability achieved.^6^ ^,^ ^7^

The lack of maintenance of the anteroposterior relationship is usually associated with growth, and is more common in those patients who were treated when they were young and who, after removal of the appliance, showed residual growth within their original pattern, either skeletal Class II or Class III. This relapse can also occur in adult patients, but it is not as common, as growth at this stage is slower.^3^ ^,^ ^5^ ^,^ ^29^

Relapse in Class II patients can also occur due to dental and periodontal aspects. Therefore, overcorrection of Class II malocclusions is indicated, since, even with the use of retention, a relapse of 1 to 2 mm is expected, especially in those cases in which mechanics with intermaxillary elastics, in a Class II direction, were used during the finalization stage.^29^

On the other hand, maintaining Class III correction is more difficult, as it is easier to retain the growth of the maxilla than the mandible. In these cases, overcorrection in the anteroposterior direction is also indicated.^29^

Male patients with Angle Class III malocclusion require special attention during the retention phase. This concern is justified, as there may be residual growth in the condyles and consequent displacement of the mandible in the anterior direction, resulting in premature contacts in the relationship between the lower and upper incisors. The solution to this condition must be analyzed individually, considering the following alternatives:

a) Remove the upper retainer to release the incisors, which, once free, will be displaced in buccal direction. The small spaces generated can be filled with resin (preferably on the distal side of the lateral incisors).
b) Remove the fixed retention bar from the anterior region of the lower arch, carry out small wears on the proximal surfaces of the incisors and/or on the mesial surfaces of the canines, in order to allow the incisors to retract.

Once the problem has been resolved, new upper and lower retainers must be installed, and the case must continue to be monitored, as millimetric changes can cause new occlusal interferences.

#### Arch length discrepancy

The discrepancy between the volume of the teeth and their respective bone bases must be identified from the beginning of treatment. Based on this, among other diagnostic components, proximal wear (stripping), distalization or dental extractions will be planned. Expansions and projections, when not correctly indicated, will lead to relapse.^6^ ^,^ ^7^ ^,^ ^30^ ^,^ ^31^

#### Tooth size discrepancy

**Figure 3:**
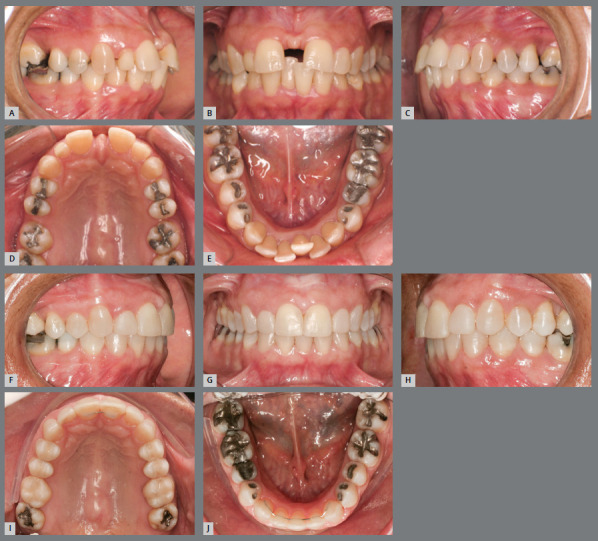
Initial **(A-**E) and final **(F-**J) intraoral images of a patient with an excess of lower anterior dental volume, narrow upper right lateral incisor and premolars. The case was resolved by extracting tooth #41 and increasing the size of the crowns of teeth #12, #14, #15, #24 and #25. The 1x1 retainer was planned to prevent the reopening of the upper midline diastema, associated with an upper removable wraparound retainer, for nighttime use. The lower occlusal view **(J)** shows the 3x3 retainer, bonded to all teeth, recommended in cases of extraction of a lower incisor.

The assessment of tooth size discrepancy, or Bolton discrepancy, reveals in which arch there is excess or lack of dental structure, whether it is in the anterior or posterior region, and its size, in millimeters. Excess dental structure in the anteroinferior region can be resolved by wear of the proximal surfaces of the teeth or by extracting an incisor. And dental volume deficiency can be corrected with proper distribution of spaces and reshaping by addition of restorative material (Fig 3). If not properly diagnosed, problems with intercuspation, overjet and overbite will occur, compromising the stability of the treatment.^3^ ^,^ ^6^ ^,^ ^7^

#### Contact points

The contact points favor the retention of tooth movements performed. Its absence leads to rotations, with the anteroinferior region being the most affected. Aiming at stability, it has been suggested that in this region, small proximal wears should be performed to create contact areas instead of contact points. Misalignment of the lower incisors is responsible for the largest number of relapses in orthodontics, and has multiple causes, such as: mesial force of posterior teeth, residual growth of the mandible, periodontal forces, occlusal factors or dental anatomy.^6^ ^,^ ^7^ ^,^ ^24^ ^,^ ^32^

### VERTICAL FACTORS

#### Leveling of marginal ridges

The leveling of the marginal ridges must be achieved by aligning and leveling the teeth. Therefore, vertical bonding errors must be corrected as soon as they are identified, so that the correct position of the teeth is maintained throughout the treatment. Furthermore, leveled ridges indicate correct axial inclinations and root parallelism of the teeth, providing greater orthodontic and periodontal stability.^6^ ^,^ ^7^ ^,^ ^14^ ^,^ ^31^ Upon treatment onset, special attention should be dedicated to teeth restored or rehabilitated by prostheses, since these were performed in the original malocclusion and, therefore, may have compensated an incorrect tooth positioning. 

#### Open bite

Anterior open bite may be associated with dental, skeletal pattern and/or muscular changes, either due to the presence of extrinsic or intrinsic habits. Most of these cases require transdisciplinary treatment, involving other health professionals, so that all stomatognathic functions are performed correctly and dental and muscular balance is established.^29^ ^,^ ^30^

During treatment, special care must be taken in diagnosing the etiological factor. Open bite may be caused by the thumb or pacifier sucking habit, by forward tongue positioning, or if the patient is a mouth breather. The association of factors, in addition to complicating the treatment itself, can compromise the maintenance of results achieved.^33^

Special attention must be dedicated to mouth breathers, as, due to the permanent open mouth position (to perform the vital respiratory function), they develop hypotonic muscles, which favors relapse, due to the extrusion of posterior teeth. In these cases, the mouth breathing disorder must be treated in combination with an otorhinolaryngologist and a speech therapist, to adapt the muscles in the region.^6^ ^,^ ^7^

**Figure 4:**
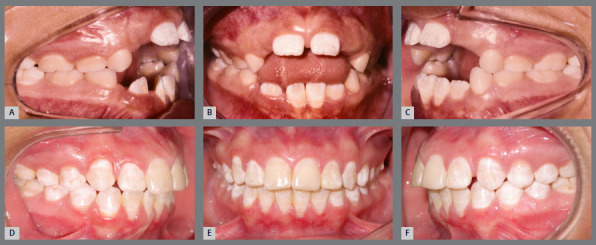
Anterior open bite, caused by thumb sucking habit and anterior tongue posture, treated with a fixed palatal bar containing active tips. Then, a removable acrylic plate with grid was used as retainer.^33^

Correct diagnosis and removal of the etiological factor will determine the success of the treatment. The habits of thumb sucking, pacifier use, and anterior tongue posture should be addressed by the orthodontist, when necessary with the support of speech therapy (Fig 4). 

Concerning the retainers, an acrylic plate with palatal grid and/or hole may be indicated (Fig 10), which act as reminders of the correct tongue posture.^6^ ^,^ ^7^ ^,^ ^30^ ^,^ ^31^

#### Increased overbite

**Figure 5:**
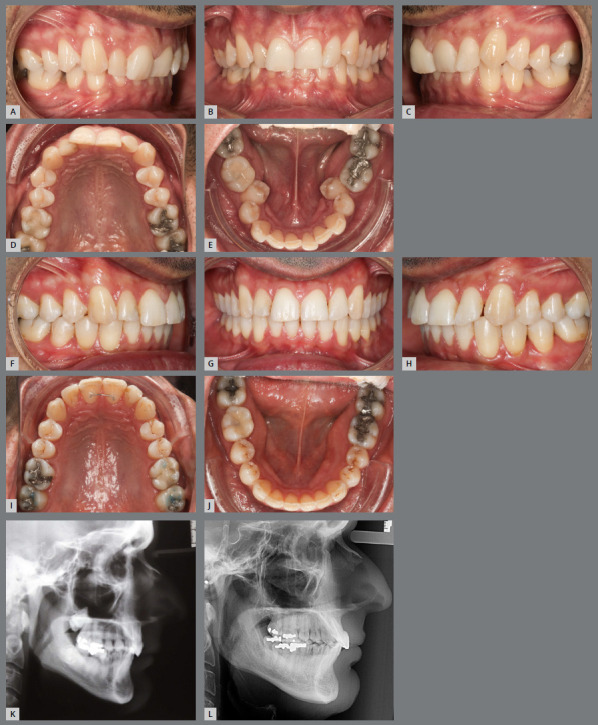
Initial images of a patient with Angle Class II, division 2 malocclusion, increased overbite, with lingually inclined upper and lower incisors **(A-E**); and after orthodontic treatment **(F-**J), with corrected axial inclinations of the posterior teeth. The initial and final lateral cephalometric radiographs **(K,**L), demonstrate the correction of the axial inclinations of upper and lower incisors.

Concerning the increased overbite, at the end of treatment the teeth must present correct buccolingual inclination in relation to their respective bone bases, and light contact of the lower incisors with the upper ones (Fig 5). If the contact in the anterior region is intense (fremitus), the upper incisors may protrude; and, if it is non-existent, it will lead to extrusion of the lower incisors. Furthermore, if the incisors are upright at treatment completion or there is no passive anterior stop in the upper retainer, there will be a greater chance of relapse of vertical malocclusion, due to extrusion of lower anterior teeth.^6^ ^,^ ^7^ ^,^ ^29^ ^,^ ^31^ ^,^ ^34^

The importance of muscle assessment should be highlighted, especially in patients with brachyfacial pattern, in which the strong masseter muscles can influence the overbite relapse, as well as some parafunctions causing overwork of muscles in the region. In these cases, associated treatments to adapt the muscles are indicated. 

### TRANSVERSE FACTORS

#### Intercanine distance

It is already known that the intercanine distance must be maintained throughout orthodontic treatment, and that changes in this distance are directly related to the instability of the implemented therapy. An exception should be considered when the canines are very inclined to lingual or when a premolar is extracted and the canine is retracted towards the wider region of the arch.^3^ ^,^ ^4^ ^,^ ^6^ ^,^ ^7^ ^,^ ^10^ ^,^ ^30^ ^,^ ^31^

#### Dental arch shape

Maintenance of the individual shape of the lower arch is mandatory in all orthodontic treatments. This is because it has already been elucidated, in several studies, that changes in the shape of the lower arch are very unstable. Changes to the upper arch are acceptable, provided they aim at promoting a good relationship with the lower arch. For that purpose, orthodontists should always choose to position the teeth using torque adjustment, rather than lateral dental inclination.^10^ ^,^ ^31^ It is important to keep in mind that the shape of the dental arch matches the adjacent muscles, representing the balance zone between tongue, lips and cheeks.^6^ ^,^ ^7^ ^,^ ^31^

#### Transverse problems

The lack of adequate diagnosis of the actual discrepancy between the maxilla and mandible can result in adverse periodontal response, unstable dental camouflage, or compromised dentofacial aesthetics. Adults who present a discrepancy between the bone bases of the maxilla and mandible, and seek long-term stability, without deleterious effects on the periodontium, should undergo surgically-assisted rapid maxillary expansion. Children and adolescents can use less invasive treatments - such as disjunction of the midpalatal suture - to correct this discrepancy and, thus, ensure long-term stability.^13^

**Figure 6:**
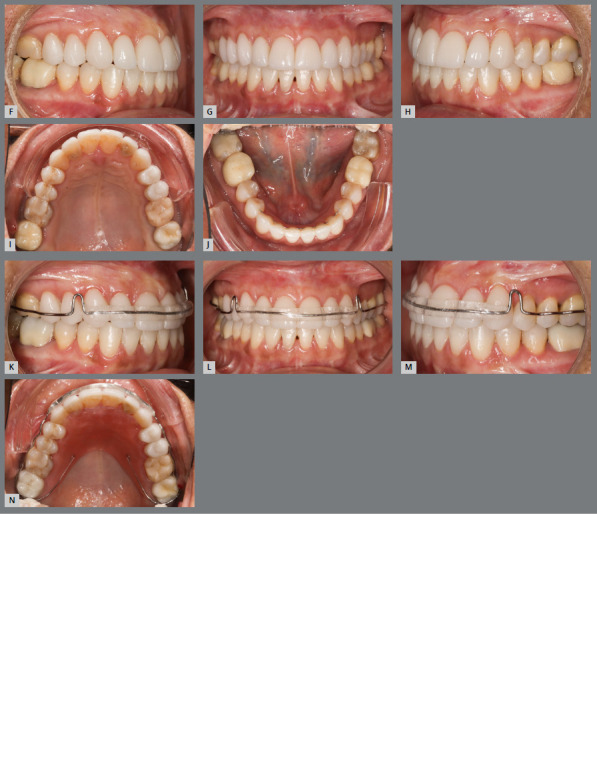
Patient with posterior and anterior crossbite. Treatment was conducted with an orthodontic and surgical approach. The upper arch was corrected with dental expansion and correction of the lingual inclinations of the posterior teeth **(F-J)**. Lower 4x4 fixed retainer **(J)**; removable upper retainer, with lateral acrylic limits extended to the palatal surfaces of the posterior teeth, for stabilization in the transverse direction **(K-N)**.

Regardless of whether there was upper dental expansion or palatal disjunction, the posterior teeth must be retained in this new position, to avoid relapse due to pressure from the buccinator muscles.^6^ ^,^ ^7^ ^,^ ^31^ In this case, retention with acrylic extension partially covering the palatal surface of the teeth is recommended (Fig 6).

### INTRINSIC FACTORS

Deleterious habits, post-treatment residual growth, and neuromuscular coordination are three intrinsic patient-related factors that are strongly related to long-term stability.^6^ ^,^ ^7^ ^,^ ^31^

Deleterious habits, or addictive habits (such as thumb or pacifier sucking, mouth breathing and forward posture of the tongue at rest) can undermine the stability of the treatment, depending on their intensity, duration and frequency.^6^ ^,^ ^7^ ^,^ ^13^ ^,^ ^31^ When the muscles are out of balance with the oral environment, due to harmful habits, it is impossible to keep the teeth in stable positions. These habits must be eliminated as soon as possible, before corrective orthodontic treatment, to prevent relapses and for the treatment to be completely successful. The orthodontist must explain to the patient why these habits are being removed, as their interruption is effective in 90% of cases, with or without the aid of devices and/or transdisciplinary treatments, either with an otorhinolaryngologist and/or speech therapist.^9^

There is controversy in the literature regarding residual growth and long-term stability. What is known is that patients with an unfavorable skeletal pattern, Angle Class II or III, must be carefully treated and monitored, aiming to avoid the need for new treatment, whether compensatory or surgical.^6^ ^,^ ^7^ ^,^ ^31^

Patients with problems in neuromuscular coordination may present instability problems, due to the absence of well-defined maximum intercuspation, the presence of dual bite, lack of tongue control and lack of treatment compliance.^6^ ^,^ ^7^ ^,^ ^30^ ^,^ ^31^

### FACTORS OF FINAL OCCLUSION

The fundamental concept of dentition dimension should be considered during the treatment plan, as it has a major impact on stability. Four limits of the dentition must be respected: anterior, posterior, lateral and vertical.^20^ ^,^ ^35^ ^-^ ^38^

Respecting the anterior limit means planning the position and inclination of the incisors in relation to their respective bone bases and the harmony of the face. Its long-term stability will be a consequence of well-positioned teeth in a balanced face. The lateral limit of the dentition is given by the shape of the arch, which tends to return to its original condition after the completion of corrective treatment. The intercanine and intermolar distances and arch shape are the best guides for the future stability of the final occlusion.^20^

**Figure 7:**
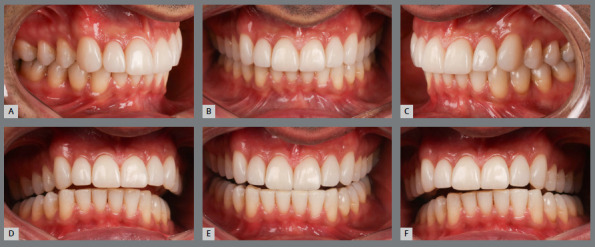
Images of the patient in Figure 4, after resin restorations, to rehabilitate function and aesthetics; with MHI and appropriate canine and incisor guidances.

Besides respecting the four limits of dentition, the occlusion must be stable and functional, i.e., it must have a correct distribution of occlusal contacts in maximum habitual intercuspation (MHI); axial or near-axial masticatory load; adequate vertical dimension and functional free space; and contacts allowing closure and excursive movements (canine and incisor guidances) without interference (Fig 7). To establish this balance of occlusion, the following may be necessary in some cases: ​​selective wear; additions, through restorations or prostheses; orthodontic/orthopedic movement, and orthognathic surgery; or a combination of these resources.^39^

The best and most conservative occlusal adjustment is undoubtedly achieved by orthodontic treatment. Selective wear can be used (before, during, or six months after completion of treatment) as a means of facilitating movement, reducing occlusal trauma and parafunction, or improving final intercuspation. It is important to emphasize that occlusal adjustment should not be used as a substitute for tooth movement or to solve professional limitations. It is also worth highlighting that occlusal stability alone does not guarantee the absence of relapse; musculature and periodontal health are also important for long-term treatment stability.^39^

### INADEQUATE ORTHODONTIC DIAGNOSIS AND TREATMENT

Orthodontic diagnosis and treatment also influence relapse and can be considered the sixth factor. However, this is the biggest and most preventable cause of relapse, since diagnosis and treatment are two of the only factors that may be controlled by the orthodontist.^9^ The final position of the teeth must be planned based on numerous aspects already explained above.

## RETENTION DEVICES

Retainers must maintain the shape of the dental arch, stabilize the anteroposterior and vertical relationships, in addition to preventing the relapse of tooth rotations. Orthodontic retainers can be removable (acrylic plates with clasps; thermoplasticized) or fixed.^8^ ^,^ ^40^

### REMOVABLE APPLIANCES

#### Acrylic plate with clamps

Dr. Charles A. Hawley (1919), then an orthodontist in Washington (D.C., USA), published details of its retainer in the International Journal of Orthodontics, inspired by the device designed in 1906 by R. D. McBride, from Dresden (Germany). The referred device was designed with buccal wire containing two loops and Adams retention clips on the first molars. The device was designed with a buccal wire containing two loops in the anterior region and Adams retention clasps on the first molars. When applied to the lower arch, he recommended occlusal clasps between the lingual cusps of first molars to prevent the posterior plate portion from moving toward the gingiva.^41^

**Figure 8:**
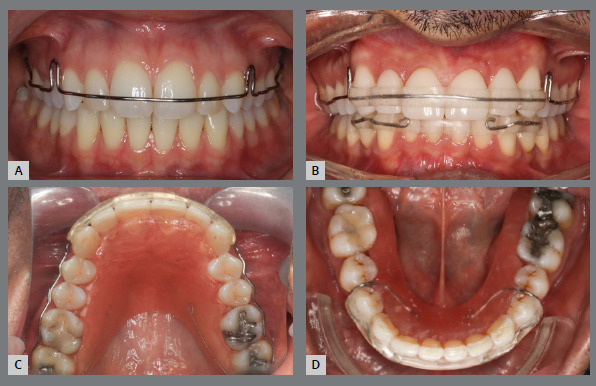
Upper wraparound retainer **(A)**, with the addition of an acrylic strip in the anterior region **(B, C)**; and lower removable retainer with acrylic strip over the wire **(D)**.

The continuous arch acrylic plate, known as the Begg Arch or Wraparound, was designed by the pioneering Australian orthodontist Percy Raymon Begg (Fig 8A). Unlike the Hawley arch, it contemplates the vestibular of the anterior and posterior teeth up to the last erupted molar, leaving the premolars and molars free, in order to allow the occlusion to settle and improve intercuspation.^42^ The 0.032-in steel wire that contours the buccal surface of teeth must have bilateral loops that can be handled for adjustments. 

**Figure 9:**
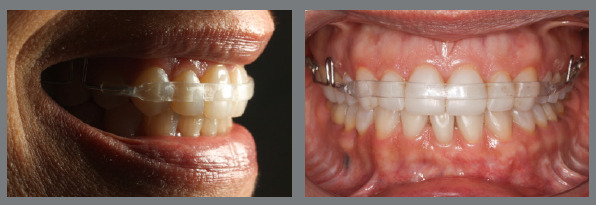
Wraparound retainer constructed with steel wire segments (0.032-in) in the posterior region and an acetate strip adapted to the incisors and canines.

It is advisable to incorporate an acrylic band over the wire (resource suggested by Simeon Hayden Guilford), on the buccal surface of the upper incisors and canines, in order to increase the contact area and provide better buccal and vertical retention of the anterior region^43^ (Fig 8B, 8C). In collaborative patients, this resource can also be used in the lower arch, replacing the fixed bar (Fig 8D). The stainless-steel wire in the anterior region can be replaced with an acetate or fiberglass band,^44^ providing a more aesthetic retention, as requested by some patients^6^ ^,^ ^7^ (Fig 9).

**Figure 10:**
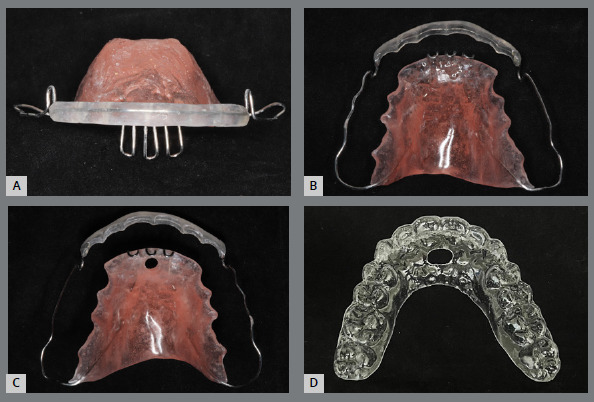
Removable devices for education and control of tongue posture: wraparound with palatal grid **(A,B)**, with grid and hole in the acrylic (C); and acetate plate with hole in the anterior region (D).

The stability of orthodontic treatment in patients with vertical problems deserves special attention during retention planning. For patients with increased overbite, a passive acrylic stop should be added to the anterior region of the upper plate (Fig 8C).^8^ ^,^ ^44^ For patients with open bite, it is advisable to install a palatal grid and/or hole in the acrylic in the palatal region, which act as reminders to educate tongue posture (Fig 10). In patients with short face and strong muscular pattern, Santos et al.^45^ advocate the use of active anterior stop, with the aim of neutralizing the harmful effects of the facial and masticatory muscles activity. For individuals with a skeletal open bite who have remaining growth potential after completing orthodontic treatment, Lima and Oliveira^46^ recommend active retention with stops in the premolar and molar region, until the end of growth, with the purpose of reducing the magnitude of vertical growth. 

For patients who wish to maintain, over time, the dental positions achieved with corrective orthodontic treatment, based on our clinical experience, we recommend the nighttime use - for life - of the removable upper retainer (Fig 8A-C), to prevent dental arch contraction resulting from the changes that occur with the aging of the tissues surrounding the teeth. Once the width of the upper arch is maintained, the intercuspation of the posterior teeth also prevents the action of time from promoting contraction of the posterior segments in the lower arch. 

### THERMOPLASTICIZED PLATE

**Figure 11:**
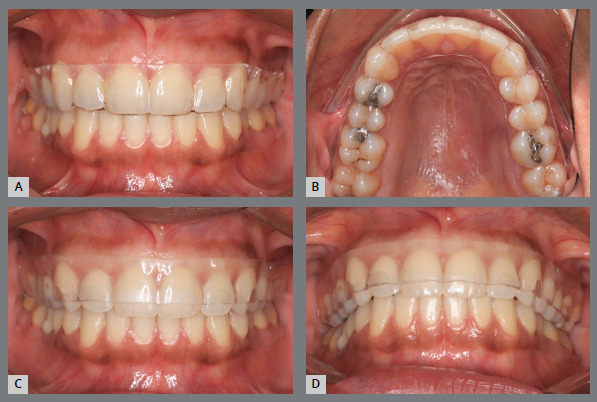
1-mm **(A,B)** and -2mm thick **(C,D**) acetate plates.

Thermoplasticized devices, also known as acetate plates, are well accepted by patients, as they have advantages such as: quick and easy fabrication and can be installed on the same day as the fixed appliance is removed; greater comfort; better aesthetics; low cost; easy cleaning; and less concern about periodontal health (Fig 11A, 11B). On the other hand, they depend more on the patient’s responsibility for their use, and it is up to them whether or not they want to maintain the results obtained with the corrective treatment. The device must not be used during feeding, whether solid or liquid, so that the material does not suffer deformation and/or pigmentation over time.^6^ ^,^ ^7^ ^,^ ^8^ ^,^ ^44^ ^,^ ^47^ ^-^ ^55^ Thermoplasticized plates, as well as acrylic plates with clasps, can be used in both arches. 

It is important to highlight that the acetate plates prevent the vertical movement of the teeth, which is desirable after removal of corrective appliances, to improve the intercuspation of the posterior segments. They function as bite-blocks, preventing occlusal settlement, which can generate intrusion of the teeth or even cause a posterior open bite.^40^ ^,^ ^51^ ^,^ ^52^ It is known that the lack of intercuspation is a risk factor for orthodontic relapse. Aiming to favor the contact of the posterior teeth, the device designed by Dr. Osamu Yoshii represents a modification in the acetate plate (2-mm thickness), with a cutout, leaving the occlusal surfaces of the teeth free^53^ (Fig 11C, 11D). This resource can be used in the upper and lower arches.

### FIXED APPLIANCES

**Figure 12:**
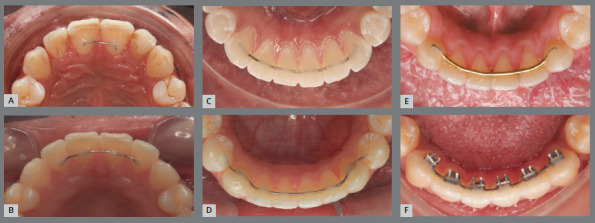
Fixed upper and lower retainers: with 0.020-in steel wire, bonded to the central incisors (1x1, in A) and bonded to the upper central and lateral incisors (2x2, in B); C) with 0.020-in steel wire, bonded to all lower anterior teeth (3x3); D) with 0.020-in steel wire, bonded up to the second premolars (5x5); E) with 0.032-in steel wire, fixed only to the canines (3x3); F) lower bar with spurs, bonded from canine to canine (over Titanium Lingual Retainer Wire tape, Reliance Orthodontic ).

Fixed retainers effectively prevent post-treatment dental changes in the anterior region of the dental arches, caused, in the short term, by relapse; or, in the long term, by the natural aging process.^6^ ^,^ ^7^ ^,^ ^14^ ^,^ ^55^ The canine-to-canine bar (3x3) is considered the gold standard as a lower retainer, because it effectively prevents the relapse of crowding of the lower incisors and the decrease of the intercanine distance, being almost socially invisible, in addition to not depending on collaboration of the patient^55^ ^-^ ^58^ (Fig 12).

In the lower arch, it is important to extend the fixed bar to the second premolars, when first premolars extractions are performed (in addition to obtaining root parallelism of the teeth adjacent to the extraction areas); and, in cases of intrusion of incisors and canines, indicated for the correction of increased curve of Spee, to avoid relapse of the aforementioned vertical movement of the anterior teeth.

In more serious situations of anterior and/or low tongue posture, we learned from Dr. Jorge Faber how to attach spurs to the lower retention bar (Fig 12E).

Medina et al.,^59^ when evaluating the impact of upper and lower fixed retainers on patients’ quality of life, concluded that: initially, only the upper fixed retainer had a negative impact, a situation that improved over time - in the long term, the presence of fixed bars did not bother patients, but the occurrence of fracture or detachment of these retentions did.

It is worth mentioning that the success of this type of retention depends on a correct adhesive technique - since there is a risk of detachment or fracture of the wire, with consequent post-treatment dental changes, related to distortion or residual activity of the wire - and also on the professional monitoring in relation to periodontal health.^55^ ^,^ ^56^ ^,^ ^60^

The 3x3 lower fixed bar, with straight stainless-steel wire with round section, should always be fixed above the interdental papillae (using as reference the union of the incisal and middle thirds of the incisors). Thin steel wire (0.020-in) can be used, bonded to the anterior teeth, or thicker steel wire (0.032-in) bonded only to the canines.^8^ In addition to these, Figure 12 shows different protocols (1x1, 2x2, 3x3, 5x5) and wire thicknesses indicated for midline diastemas, generalized diastemas, rotations and severe crowding.

The great advantage of fixed retention, bonded to all anterior teeth, maxillary and/or mandibular, is the individual retention of the teeth, especially when the initial malocclusion presents great dental crowding. However, there are disadvantages as the difficult cleaning and higher risk for accumulation of bacterial biofilm and calculus.^44^ ^,^ ^52^

After defining the contour, NiCr metal alloy retention bars must undergo tempering heat treatment, to eliminate residual stresses that occur during conformation and increase the wire capacity to resist changes in its final shape, avoiding fractures.^61^

Many professionals advocate the use of 0.0215-in coaxial wire as a retention resource, bonded to all upper or lower anterior teeth. Currently, we do not recommend this material, as unwanted effects are occurring, such as changes in arch shape and consequent failure of tooth alignment, due to wire distortion. 

Also, we do not recommend modified retainers with multiple vertical loops, called “hygienic retainers”, because: they have a large length of wire, which favors greater accumulation of bacterial plaque and calculus; they do not allow the use of dental floss to properly clean the gingival sulcus, which is important to prevent vertical bone loss; the number of incorporated folds can have a spring action and result in unwanted tooth movements, creating spaces or changes in root torque, leading to bone dehiscence and gingival recessions.^62^ ^,^ ^63^

To facilitate the use of dental floss in the interproximal regions, but without the negative effects that hygienic retainer causes, Ribeiro et al.^64^ presented a 3x3 fixed retention bar model with V-bends in the horizontal direction. However, clinical studies need to be carried out to prove the effectiveness/efficiency of this type of retention, when compared with the 3x3 bar bonded to all lower anterior teeth.

Another fixed retainer model that has been used is the fixed bar that replaces the metal by fiberglass, which meets the stability criteria of the retention phase similar to the fixed metal bar or strip, combined to a more pleasant aesthetic component. However, despite controversies in the literature, fixed fiberglass retainers have shown greater periodontal complications and, therefore, fixed metal retainers should continue to be the gold standard in Orthodontics.^65^ ^,^ ^66^

### ASSOCIATION OF APPLIANCES

For safety, fixed and removable retainers can be used in the same dental arch, such as, for example, the 3x3 fixed bar and an overlapping acetate plate. The advantage is that, if there is detachment or deformation of the wire, the removable retainer - recommended for nighttime use - will maintain the position of the teeth until the fixed bar is reattached; in addition to functioning as a backup until the patient can be assisted for proper repair.^8^ ^,^ ^51^

### DURATION OF RETENTION

Several factors influence the duration of retention use, such as: orthodontist preference; variability of occlusal, skeletal and soft tissue relationships; and the scarcity of well-controlled scientific studies on this subject^8^ ^,^ ^51^. The period of 12 to 36 months is found in the literature to control the case and, in addition, to encourage the patient to continue using the retainer, ensuring long-term stability^9^ ^,^ ^67^ ^-^ ^71^.

**Figure 13:**
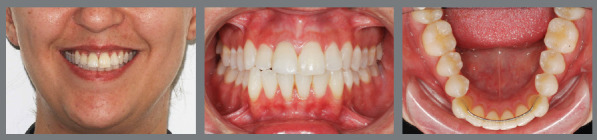
Observing the action of facial aging (which occurs slowly and gradually) on the dentition of a patient treated orthodontically for many years, it can be seen that: due to the presence of the 3x3 retainer, the lower anterior region has not changed; constriction occurred in the middle region of the upper and lower arches, a situation that could have been avoided if the patient had used the upper retainer correctly.

Since tooth movements are performed in a living organism, which is functioning and also subject to changes with advancing age, we recommend the use of retainers for life (fixed lower retainer and removable upper retainer). Once the width of the upper arch is maintained, the intercuspation of the posterior teeth prevents the action of time from promoting contraction of the posterior segments in the lower arch (Fig 13). 

Concerning the time of use of removable retainers, we advocate a minimum use of 10h/day in the first year of the retention phase and then 6 to 8h/day (nighttime use) throughout life. Over time, in the yearly patient evaluation, it is possible to change the use of removable retainers to alternate nights or three times a week. 

In case of presence of spaces, severe crowding in the anterior region (upper and lower), or in patients with periodontal disease with bone loss, it is advisable to install fixed retainers, to avoid risks and eliminate the need of patient compliance to use the removable retainer for more hours per day. 

The patient must be informed that retainers installed after completion of treatment may suffer deformation over time, and new devices need to be manufactured and installed. 

## PATIENT COMPLIANCE

Before starting orthodontic therapy, it is extremely important that the patients are aware that: retention is also part of the treatment; they will be primarily responsible for this phase; lack of collaboration will compromise the final result achieved by corrective treatment.^6^ ^,^ ^7^

Retainers can initially cause a negative impact in terms of comfort and functional limitations (such as speech, for example). However, over time, this discomfort decreases considerably.^52^

Another relevant point is the scheduling of return appointments to check the retainers. During the first year, when there is a greater likelihood of tooth movement, appointments at every four months are recommended to observe the patient more closely and reinforce use and care. From the second year onwards, yearly appointments are recommended, which are important because the retainers can be damaged.

To prevent more severe relapses, dental monitoring software is being used so that the orthodontist can remotely evaluate possible changes during the retention phase.^72^

There may be patients who never use retainers and still maintain the results of orthodontic treatment, but orthodontists are unable to identify these patients at the beginning of treatment. Therefore, everyone should be treated as if they had a high potential for relapse. And long-term use of retainers is the best way to maintain the stability of orthodontic treatment over the years.^73^

## APPLIANCES HYGIENE

It is up to the patient to follow the hygiene guidelines provided by the orthodontist and be careful with the retainers. However, the general dentist must be guided to reinforce the importance of using the prescribed retainers, besides helping to maintain good dental and periodontal health, especially of the lower fixed retainer, which, due to its location, tends to accumulate more bacterial plaque and dental calculus.^8^ ^,^ ^44^

Removable retainers must be cleaned before use and after removal from the oral cavity, using a stiffer-bristled toothbrush, with toothpaste or mild soap. For proper cleaning, it is recommended to immerse the retainer in a container with water and a specific tablet to eliminate bacteria. 

Concerning the fixed bars, since brushing will take place in the mouth, together with the other teeth, greater care must be taken with the use of dental floss, which, in order to fulfill its function in an integral manner, must reach the proximal surfaces of the teeth and the gingival sulcus.

**Figure 14:**
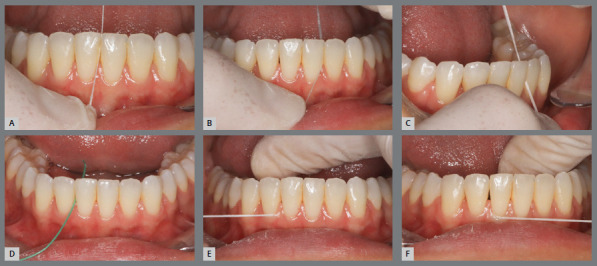
Cleaning the fixed lingual bar, with dental floss above **(A-C)** and below **(D-F**) the retention bar.

For fixed retainers bonded only to the canines, the dental floss must be passed “hugging” the mesial and distal surfaces of each of the four lower incisors and the mesial surfaces of canines. For fixed retainers bonded to all anterior teeth, dental floss must be used in two steps: above the bar (placed vertically) to clean the proximal surfaces of the region; and below the bar, using a floss threader (placed horizontally) to complement the cleaning of proximal surfaces of teeth and reach the gingival sulcus region (Fig 14). This guidance is also recommended for cleaning fixed retainers in the anterior region of the upper arch.

## BIOMATERIALS AND DRUGS - A FUTURE PERSPECTIVE

The use of some biomaterials and drugs has been studied in animals, with the aim of verifying the improvement in immediate stability after tooth movements induced by orthodontic appliances. These include carbonated hydroxyapatite associated with fibrin-rich plasma, which was administered by local injection into the periodontium of rabbits and demonstrated a significant decrease in relapse, compared to the control group - since it stimulates the proliferation of osteoblasts, accelerating the reestablishment of the alveolar bone and local periodontium.^74^ ^,^ ^75^

Similarly, simvastatin injected into the periodontium of rats after tooth movement demonstrated changes in local osteogenesis, as well as a reduction in bone resorption, reducing the possibility of relapse.^75^ ^,^ ^76^ Bisphosphanates, which have an effect on the osteoclasts activity, have also been studied for this purpose, with positive results regarding the reduction of relapse in rats after seven days of tooth movement, when injected into the periodontium.^75^ ^,^ ^77^

## FINAL CONSIDERATIONS

Orthodontic retainers are not all the same. The retention protocol must be carried out in a personalized and planned manner, taking the initial tooth positions as a reference. In the short term, retainers will prevent tooth relapse during the remodeling of the protective periodontium (gingiva and alveolar mucosa) and supporting periodontium (periodontal ligament and alveolar bone); and, in the long term, they will not allow biological tooth movement caused by residual growth and/or the natural aging of the individual. 

It is important to note that, before treatment onset, patients must be informed that they will use retainers after the end of orthodontic therapy, following the protocol determined by their orthodontist, and must understand the importance of their use. To highlight the relevance of this new phase in the stability of corrective treatment, it is recommended to schedule a specific appointment with the patient (and legal guardians, if necessary), to formalize and clarify important aspects about the new treatment stage, as follows:

» To maintain the functional and aesthetic results achieved with corrective treatment, it is essential to follow the correct use and care of the prescribed retainers.
» Teeth position may change after corrective treatment, due to: changes in the bone and gingival tissues surrounding the teeth; presence of harmful habits; influence of changes in chewing and speech, which occur over time; changes in the tissues that make up the face, due to growth, development and aging.
» Retainers installed after the completion of corrective treatment are not everlasting; rather, they may be damaged by use and must be replaced.
» Obtaining radiographic and photographic records after completing treatment is important to protect both the patient and the professional.
» The patient must return for follow-up appointments when scheduled by the orthodontist.

To establish the commitment, patients must sign a document certifying that they have received guidance on the importance of their responsibility in maintaining the results achieved in the active phase of orthodontic treatment.
